# Topical Finasteride: A Comprehensive Review of Androgenetic Alopecia Management for Men and Women

**DOI:** 10.7759/cureus.44949

**Published:** 2023-09-09

**Authors:** Akshunna Keerti, Bhushan Madke, Akshaya Keerti, Mark Joseph C Lopez, Francesca S Lirio

**Affiliations:** 1 Dermatology, Jawaharlal Nehru Medical College, Datta Meghe Institute of Higher Education and Research, Wardha, IND; 2 Professor and Head, Dermatology, Venereology and Leprosy, Jawaharlal Nehru Medical College, Datta Meghe Institute of Higher Education and Research, Wardha, IND; 3 Oncology, Meherbai Tata Memorial Hospital, Jamshedpur, IND; 4 Medicine and Surgery, Manila Central University, Manila, PHL; 5 Internal Medicine, Las Pinas City Medical Center, Las Pinas, PHL; 6 Internal Medicine, Unihealth Paranaque Hospital and Medical Center, Paranaque, PHL; 7 Internal Medicine, East Avenue Medical Center, Manila, PHL; 8 Doctor of Medicine, University of the East Ramon Magsaysay Memorial Medical Center (UERMMMC), Manila, PHL; 9 General Medicine, University of Santo Tomas, Manila, PHL

**Keywords:** topical finasteride, androgenic alopecia, genetics, efficacy, patterned hair loss

## Abstract

The most prevalent kind of alopecia, androgenetic alopecia, commonly known as male or female pattern hair loss, affects both men and women, with the frequency rising with advancing years. Even though practicing dermatologists and hair experts frequently encounter it, it might be one of the most challenging disorders to treat since choosing a course of action frequently requires a comprehensive analysis of several variables and moral judgment. Effectiveness, side effect profiles, practicability, promoting compliance, and treatment cost are the most important factors to take into account, especially given the chronic nature of androgenetic alopecia. A clinician's ability to select the optimum course of treatment for each patient may be constrained and clouded by their knowledge base, experience, and financial compensation. A search was done to find research on the effectiveness of topical finasteride therapy, including clinically pertinent case reports and papers. Only topical minoxidil and oral finasteride are now approved by the Food and Drug Administration and the European Medicines Agency for the treatment of androgenetic alopecia. Despite being effective for hair regeneration, systemic use of finasteride is accompanied by adverse effects that prevent long-term use. Investigating topical finasteride as another possible treatment plan may be fruitful. Early research on the use of topical finasteride is safe and encouraging, despite its limitations. More research on drug distribution, ideal topical strength and usage regularity, adverse effects, and application for other alopecias would aid in elucidating the range of topical finasteride use.

## Introduction and background

Dermatologists treat androgenetic alopecia (AGA), a common chronic cutaneous condition worldwide. Despite its limitations, the early study on the application of topical finasteride is positive and safe. With greater research into medication distribution, ideal topical strength and usage regularity, adverse effects, and application for other alopecias, the range of topical finasteride (FNS) utilization will be made clearer. The Food and Drug Administration (FDA) and the European Medicines Agency (EMA) have only approved topical minoxidil (MNX) and oral finasteride (FNS) as therapy options for AGA, despite the condition's high patient morbidity and prevalence [1]. Doctors may perform surgical hair transplants if no other therapeutic modalities are available while a patient is independent to choose a hair transplant over other therapeutic modalities [2].

The potential binding of dihydrotestosterone (DHT) to androgen receptors (AR) found in the hair follicle is hypothesized to have an impact on the pathophysiology of AGA. The 5-reductase type 2 enzyme in the follicle dermal papilla converts testosterone into dihydrotestosterone. DHT levels are influenced by the quantity of weak androgens, testosterone conversion, the action of the androgen-inactivating enzymes, and the quantity of androgenetic receptors. DHT levels are high, and AR expression is elevated in the dermis which is prone to AGA [3].

Clinical settings use systemic finasteride, a 5-reductase inhibitor and 4-aza-3-oxosteroid molecule, to treat benign prostatic hyperplasia (BPH) and AGA. FNS inhibits 5-reductase type 2 competitively to stop testosterone from being converted to dihydrotestosterone and significantly lower blood DHT levels. The usual terminal half-life of finasteride in men between 18 and 60 is five to six hours, but it is eight hours in men over 70. DHT levels return to normal 14 days after stopping the treatment [4]. It is expected that hair growth will stop within a year of stopping systemic FNS treatment for AGA. Male breast hypertrophy and malignancy, mastalgia, decreased ejaculatory volume, testicular atrophy, testicular hypoalgesia, decreased penile curvature, penile atrophy, sexual dysfunction, infertility, prostate malignancy, and prostate inflammation are a few of the side effects of this medication in its systemic form. Due to their sensitivity to sexually harmful side effects, male patients are generally unable to afford these side effects [5].

Topical FNS may be protective against AGA, according to animal research. In a mouse model of testosterone-induced alopecia alba, topical FNS 2 percent solution was shown to have greater follicular density and anagen: telogen ratios than fern extract (Adiantum capillus-veneris). Twenty years ago, a study revealed the initial effectiveness and safety assessment of topical FNS treatment in patients with AGA [6]. Recent research suggests that, compared to systemic medicine during the previous five years, topical FNS may be a beneficial therapeutic alternative. This study will provide an overview of previous and ongoing clinical investigations looking at topical finasteride therapy for androgentic alopecia [7].

## Review

Methods

**Figure 1:**
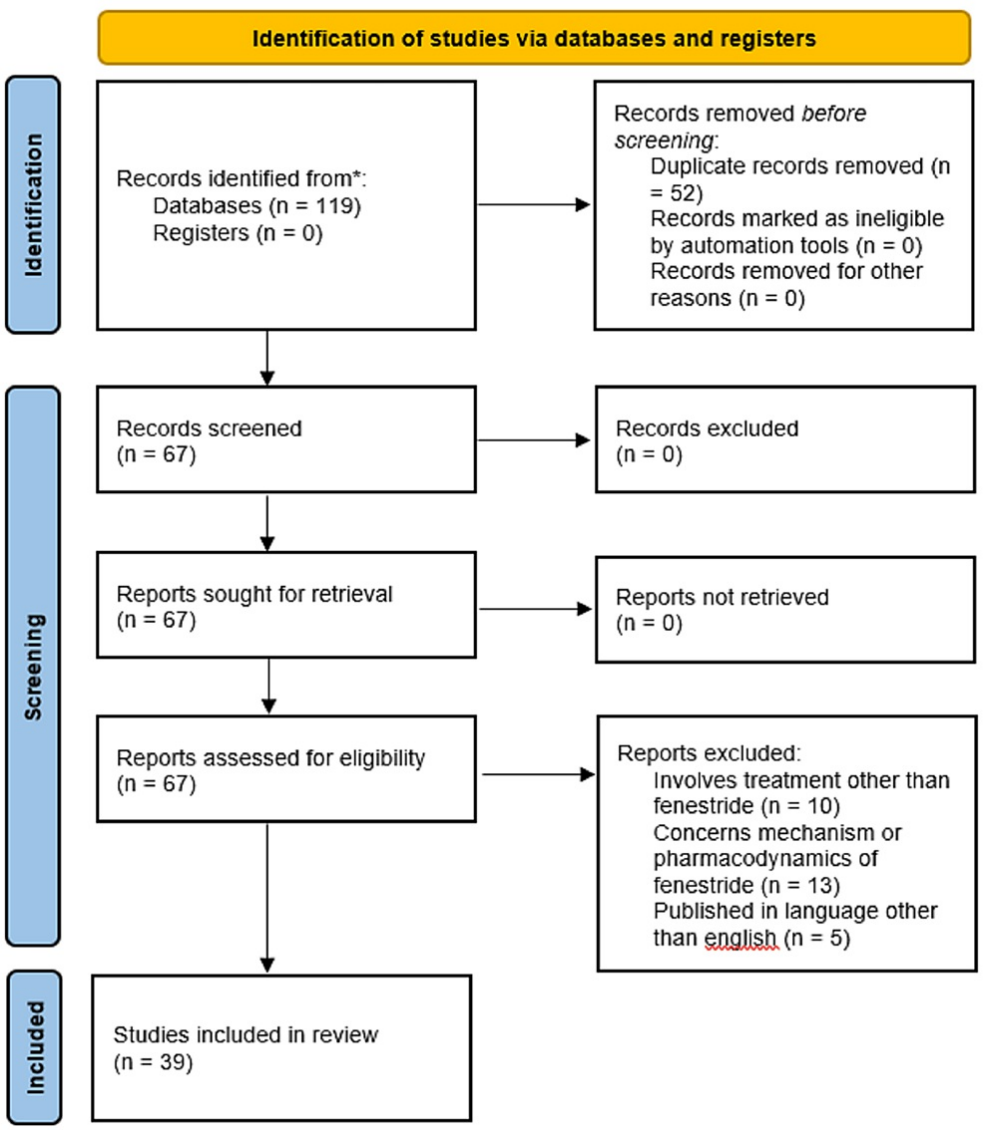
Selection criteria of articles This figure is self-made by the author

A literature search was carried out using the terms "topical finasteride," "androgenic alopecia," "genetics," "efficacy," and "patterned hair loss" in the PubMed/MEDLINE, Embase, and PsycINFO databases. Since the therapeutic use of topical FNS is the main focus of the article, clinically pertinent reviews and studies were taken into consideration. For inclusion, studies must include at least one topical FNS-using treatment group, have data on topical FNS effectiveness, and only use in vivo therapy. Studies not published in English, those that only used oral FNS as a type of therapy, and those that only looked at the pharmacodynamics or mechanism of topical FNS were all disregarded. Figure 1 below shows the selection criteria for articles.

Epidemiology

Men

The most prevalent kind of baldness, AGA, is characterized by gradual hair loss. People of any color can have AGA; however, prevalence rates differ. The majority group is assumed to be Caucasians. According to estimates, the prevalence rates of men in their 30s, 40s, and 50s in Caucasian populations are around 30%, 40%, and 50%, respectively. In India, a population-based study with 1005 participants found that males between the ages of 30 and 50 had a 58% incidence of AGA. There is evidence that oriental races have a lower incidence. The prevalence was 21.3 percent in Chinese research as opposed to 14.1 percent in Korean research. Most of the studies to date suggest that incidence increases steadily with age [8].

The most usual type or grade of presentation (as specified by the Norwood classification) has been the subject of studies that have led to various results. Type II of AGA is the most prevalent symptom, according to a thorough examination of the Indian population. A different study carried out in an Indian community found that Type II and Type III presentations were most prevalent. Type IV was the most prevalent form in the Chinese study, but type III was the most prevalent type in the Korean study [9].

Women

There is less epidemiological research on AGA in women. A Norwood study with 1006 Caucasian individuals found a total incidence of around 19%. Studies of Chinese and Korean populations found that the prevalence was only 6.0% and 5.6%, respectively, showing that, like men, Asian races are thought to be less common than Caucasians. [10]. Age also increases the frequency of AGA in women. According to medical professionals, there are a few key ways that female AGA is different from male androgenetic alopecia. Female AGA may be better described as female pattern baldness. Male and female baldness exhibit different clinical patterns, pointing to the existence of two separate entities. Research that hasn't definitively linked elevated testosterone levels to female-pattern hair loss supports this [11].

Pathogenesis

The features of AGA include vellus conversion of the terminal hair follicle and step-by-step hair follicle shrinkage brought on by modifications in the subtleties of the hair cycle. The active growing stage (anagen) in a normal hair cycle might span two to seven years. After that, a brief resting phase (telogen) lasting five to six weeks to about 100 days is followed by a brief period of regression (catagen) lasting one to two weeks. The bulk of follicular keratinocytes encounter a spurt of caspase-mediated cell death, the cessation of pigment manufacture, and the shortening of dermal papilla cells during the catagen phase, a time of involution. As a result, the dermal papillae elevate. During the telogen phase, the hair shaft converts into a club of hair [12]. The anagen phase begins again after cleansing the hair.

In AGA, the time between the anagen and telogen phases gets shorter and shorter. Since the period of the anagen phase impacts the dimension of the hair, which results in miniaturization and, eventually, a bald appearance, the maximal extent of the current anagen hair is less than that of its precursor. Numerous studies concur that androgen, as well as the interaction between the dermal papilla cells and the hair follicle, play a crucial role in the miniaturization of hair follicles [13].

Higher concentrations of DHT, 5 alpha-reductase, and AR are seen in the scalp of balding individuals. The two enzymes that transform weak androgens into potent androgens, 3 hydroxysteroid dehydrogenase (3 HSD) and 17 hydroxysteroid dehydrogenase (17 HSD), have comparable amplified action in androgenetic alopecia. The influence on the expression of genes that govern the follicular cycle increases with increasing androgen and androgen receptor concentrations [14].

In balding individuals, the dermal papillae and hair follicle crossing points signal an early end of anagen and an early entry into catagen. Reduced manifestation of anagen-preserving factors, such as the growth factors IGF-1, bFGF, and VEGF, leads to catagen. Elevated cytokine expression (TGF-1, IL-1, and TNF) encourages apoptosis. DHT has lately been shown to up-regulate the gene DKK-1, which inhibits outer root sheath cells and causes programmed cell death [15].

Another recent revelation is the recognition of the crucial role played by the Wnt/catenin signaling route in maintaining the dermal papilla cells inductive characteristics needed for hair follicle rejuvenation and hair shaft development. Androgens and ligand-activated AR may have a negative impact on the Wnt/catenin signaling route. Androgens obstruct the process by amplifying the expression of glycogen synthase kinase 3 (GSK 3) [16].

The functions and mechanisms of hair follicle stem cells in androgenetic alopecia are still unknown. Even though KRT15 (hi) stem cells are still present in bald scalps, it is believed that the change of hair follicle stem cells into CD200-rich and CD34-positive progenitor cells, both of which are necessary to preserve normal follicular action, is faulty [17]. Androgenetic alopecia is a complicated disease triggered by interrelation among numerous genes and environmental factors. Because of the widespread symptomatology and high prevalence of the disease, a polygenic mode of inheritance has been discovered [18].

The X-chromosome AR/EDA2R and the PAX1/FOX A2 locus on chromosome 20 are the two main genetic risk loci. Recent research has recognized the HAD C9 gene on chromosome 7 as a unique susceptibility locus [19].

The gene for the androgen receptor: The androgen receptor regulates a cell's sensitivity to androgen. The quantity of androgen that hair follicles may receive is regulated by the AR gene. The polymorphism known as Stu 1 has the strongest correlation with AGA out of all the identified variants in the AR gene. A few other genes, including 5'-reductase, aromatase, estrogen receptor, and IGF-2, could not have their relationships shown beyond a shadow of a doubt. Understanding the role of the Y chromosome requires a more extensive genomic study [20].

Hair follicle inflammation and environmental factors: The relationship between these results and follicular inflammation has been shown by numerous studies. The procedure is slow, undetectable, and indolent, in contrast to the inflammatory and destructive procedure seen in conventional scarring hair loss. Agents like Propionibacterium sp., Staphylococcus sp., Malassezia sp., or Demodex may cause inflammatory reactions. As an alternative, irritation from cosmetics and personal care items, pollutants, and UV-induced actinic damage may cause keratinocytes to produce nitric oxide and radical oxygen species in response to chemical stress [21].

Etiology

Each hair grows asynchronously and individually from a hair follicle according to a cyclical process known as the hair growth cycle. This cycle consists of four stages: (1) The longest period, known as the anagen or growth phase, lasts for two to seven years; (2) the catagen or transition stage, which is composed of hair follicle involution brought on by apoptosis and lasts for about two weeks; (3) after removing the old hair for 12 weeks, the telogen or resting period begins; and (4) the telogen hair's releasing phase is known as the exogen phase. The hair follicle shrinks, which is a defining trait of androgenetic alopecia. In the middle of the late catagen and early anagen phases, it affects the dermal papilla and dermal sheath, giving rise to a smaller follicle and a shorter anagen phase. The abnormality is frequently permanent, even if partial regrowth and some miniaturization reversals are occasionally possible [22].

**Figure 2:**
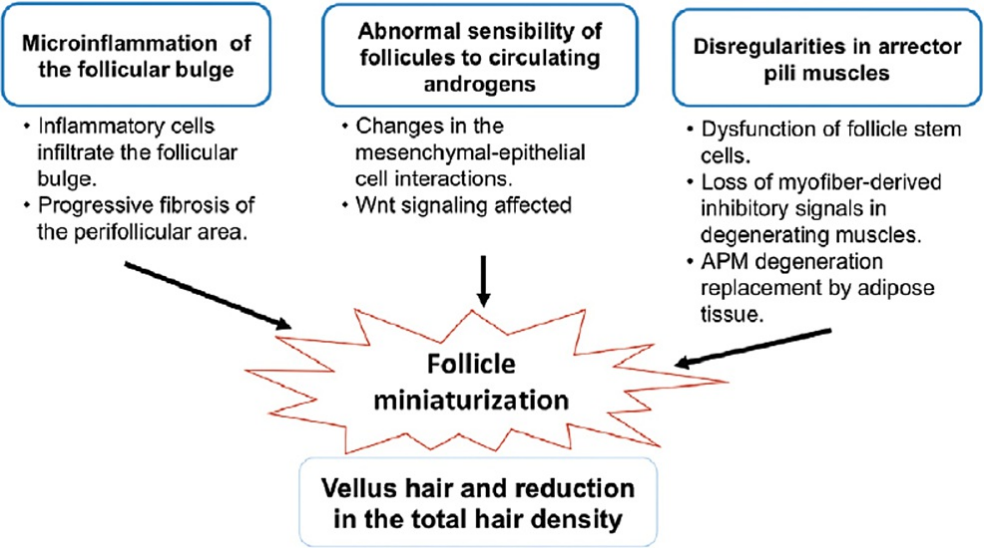
The pathophysiology of androgenetic alopecia is influenced by a variety of variables. Miniaturization of hair follicles, which is clinically known as vellus hair, is caused by microinflammation, aberrant androgen sensitivity, and abnormalities in the arrector pilli muscles. Derived from [23] by CC-BY-NC-SA 4.0 licence APM: arrector pili muscle

The action of androgenic hormones and genetic susceptibility are linked to the illness. It displays a configuration of familial aggregation, but because it can arise in a number of family members without monogenic inheritance, it is recognized as a complicated characteristic. Two studies showed a heritability of 0.81, indicating that heredity probably has a big impact on how the illness develops and manifests [22]. Figure 2 below shows how the pathophysiology of androgenetic alopecia is affected.

The increased sensitivity of scalp hair follicles to androgens in the circulation, caused by an increase in androgen receptors, has been thought to cause androgenetic alopecia. The enzymes 5-alpha-reductase, types 1 and 2, catalyze the transformation of testosterone into 5-alpha-dihydrotestosterone. Both isoforms, whose expression varies according to the region of the body, are hypothesized to affect the metabolism and action of androgen. A study found that 5-alpha-reductase type 1 expression was higher in hair follicles, signifying the crucial role of this enzyme in androgen-regulated hair formation [24].

Androgens alter the interactions between mesenchyme and epithelial cells within the follicle, which affect the size and make-up of dermal papillae, the activity of keratinocytes and melanocytes, and the growth of hair. The Wnt signaling pathway controls dermal papilla cells and may be crucial in how androgen influences hair growth [25]. However, the fundamental biochemical processes underpinning androgen-related effects are still completely unclear.

The primary clinical presentation of AGA includes a reduction in total hair density (hair/square centimeter) as well as the replacement of terminal hair by vellus hair (defined as hair 30 m in diameter and 30 m in length; transitional hair width between 30 and 40 m; terminal hair >40 m). Histologically, it is also possible to find vellus follicles in lieu of terminal follicles, a perifollicular infiltration of macrophagic cells, a rise in the number of sebaceous glands, and dermal thinning [26].

There is still disagreement about whether the action of androgens is the primary cause of follicle miniaturization. According to an androgen study, which supports the hypothesis that androgens play a role in AGA, miniaturization in individuals using finasteride can be reversed in just one hair cycle. The miniaturization found in patterned hair loss may be directly related to a decline in the quantity of cells and, consequently, the dimensions of the dermal papilla. A study claimed that when stem cells from the arrector pili muscle are employed to support the follicle, the stem cell mass in the bulge or dermal sheath is activated. The absence of communication between the bulge and the arrector pili muscle in miniaturized follicles induces miniaturization by interfering with the action of the follicle's stem cells [27].

5-alpha-reductase inhibitors

The genetic susceptibility of the hair follicles to the hormone dihydrotestosterone, which is created when testosterone is transformed to its active form by the enzyme 5-alpha-reductase, is one of the etiologic components of androgenetic alopecia. There are two different 5-alpha-reductase types in humans. Type I is mostly found in the scalp, liver, and skin, whereas type II predominates in the prostate, genitourinary system, and hair follicle.

FNS, a type II 5-alpha-reductase inhibitor, and dutasteride, a type I and type II 5-alpha-reductase inhibitor, are two medications that block the 5-alpha-reductase used in androgenetic alopecia [28].

Oral FNS 1 mg twice a day is optimal for AGA male patients above the age of 18 who have mild to severe AGA. Although it might not be evident in some men until 12 months, the response to therapy should be evaluated at six months. Taking 0.5 mg of oral dutasteride daily is another option. Though there are not adequate trials to assess its effectiveness compared to FNS [29]. Less research has been done on the use of finasteride in females. Pregnancy is a contraindication to using finasteride. Erectile dysfunction, decreased libido, and gynecomastia are rare but recorded side effects. Furthermore, finasteride lowers prostate-specific antigen (PSA) levels. Monitoring of PSA levels should be considered if therapy begins after the age of 45. For a correct interpretation of the test, the PSA levels should be doubled to account for the drop brought on by finasteride [30].

It is ineffective in postmenopausal females, according to studies. Pregnancy is not advised when using it. Finasteride is ineffective for AGA when used topically. Studies on both humans and animals have shown that combined treatment with MNX 2 percent and FNS 1 mg is superior to either FNS or MNX monotherapy. Finasteride added to a hair transplant is also thought to be more successful than a hair transplant alone [31].

The hair follicle's stem cells are situated in the bulge, where they often substitute among the active and dormant stages to maintain the stem cell population and produce new hair follicles. A study discovered that the FOXC1 gene was significantly expressed in active murine hair follicle stem cells, maintaining stem cell adhesion and assisting the transition to the quiescent state. A transcription factor called FOXC1 controls how the embryo and the eye grow. The signaling triggered by Nfatc1 and bone morphogenetic proteins helps to maintain and expand hair follicles [32].

By keeping each hair follicle in a follicular unit together at the isthmus, the arrector pili muscle significantly contributes to preserving follicle integrity. Arrector pili muscle deteriorates as a result of androgenetic alopecia, according to studies, and is replaced by adipose tissue. It is unclear how follicle miniaturization and hair loss are mechanistically connected to arrector pili muscle degeneration and fat infiltration. Follicle miniaturization has been connected to the abnormal growth of the residual adipocyte-producing progenitor cells in the arrector pili muscle. Muscle tissue with ectopic fat deposition has mesenchymal progenitor cells, which may be a clue that failing muscles lack the myofiber-produced inhibitory signals that prevent cellular adipose development. A study claimed that female pattern hair loss (FPHL) and male pattern hair loss (MPHL) both have growth-restricted (dormant/kenogen) nonvellus hair follicles that can be reactivated through medication. These observations support the significance of follicular miniaturization in the pathophysiology of androgenetic alopecia. More research is required to fully clarify the function of follicle miniaturization in androgenetic alopecia.

According to an investigation into the function of microinflammation in the pathogenesis of AGA, the inflammatory cell infiltrate in the follicular bulge results in progressive fibrosis of the perifollicular zone, which harms follicular stem cells, obstructs the normal hair cycle and ultimately results in hair loss [33].

Genetics and androgenetic alopecia

Genetic predisposition is a significant factor in the etiology of androgenetic alopecia, even though androgens serve as its mediator. Androgenetic alopecia has a complicated genetic makeup. Candidates for androgenetic alopecia are the genes AR and 5-alpha reductase.

Numerous studies have focused on genes linked to the sex-steroid pathways as a result of observations of different points of sex-steroid receptor expression and metabolizing enzymes between the balding and occipital portions of the scalp. Facts suggest that balding follicles on the exact scalp exhibit higher levels of 5-alpha reductase enzyme and androgen receptor expression than nonbalding follicles. This is because of the expression of the different genetic factors that are responsible for 5-alpha reductase type I and type II and androgen receptors in these areas [34].

The androgen receptor gene is thought to contribute up to 40% of the total genetic risk, which is a large level of risk for a single gene. The AR gene's relationship to male pattern baldness has been the subject of several investigations. Single nucleotide variants, copy number variations, and triplet repeats are among the polymorphisms that have been considered in androgenetic alopecia. A study examined the allelic frequencies of the Stu restriction site with the two polymorphisms, the CAG and GGC triplet repetition polymorphisms, in exon 1 of the androgenetic receptor gene. This study found that 98.1 percent of younger and 92.3 percent of older boys with AGA had a Stu restriction polymorphism, in contrast to 76.6 percent of non-bald controls [35]. Male pattern baldness is caused by a polygenic process, and males with baldness had fewer trinucleotide repeats combined with them more frequently (p = 0.03), signifying that these markers are a close functional variation involved in this process. A study reproduced this research in 2005 and demonstrated that the polyglycine GGN triplet repeat was the most likely candidate for AGA. Later, a study discovered that these polyglycine repeats do not increase one's risk of developing AGA, and investigations of copy number changes in AR also support this conclusion [36].

The X-chromosome AR and EDA2R genes were demonstrated by a study to be closely related to AGA. When compared to the SNP at rs1385699 in the EDA2R gene, which showed the strongest relationship signal (p = 3.9 10-19), the variation at rs6152 in the androgenetic receptor gene revealed less relevance (p = 4.17 10-12). Statistics show that while the function of EDA2R in male and female pattern hair loss is uncertain, linkage disequilibrium appears to be the cause of the linkage between markers in EDA2R and AR. The X chromosome's location of the androgenetic receptor and the substantial linkage signal of the EDA2R gene highlights the maternal lineage's role in the inheritance of both male and female-pattern hair loss. The findings highlight the importance of the AR gene, which has been linked to a greater possibility of AGA in males and has been supported by a number of previous studies. A gene effect has yet to be proven in women [37].

Epigenetic changes in androgenetic alopecia

The fact that histone or DNA methylation-based epigenetic mechanisms, in which the genomic DNA sequences are unaffected, influence the availability of inheritable factors to the transcriptional apparatus and genetic factor regulatory processes has been established. However, little research has examined epigenetics's impact on the physiology of hair follicles and the etiology of androgenetic alopecia [38].

The methylation patterns of various genes, notably the AR gene, have been the focus of recent research. A study looked at the androgenetic receptor gene's methylation patterns in both the afflicted and unaffected occipital hair follicles. They found that the occipital follicles had higher levels of AR gene methylation, which may protect them from miniaturization and hair loss. Different studies found that animals missing DNA methyltransferase 1 (DNMT1) expression over the integumentary system displayed balding traits. It is possible that DNMT1's activity is essential for the beginning of AGA, given that this gene is involved in establishing and modulating methylation patterns for tissue-specific cytokines [39].

Discussion

There have only recently been a few prospective studies, randomized controlled trials, and retrospective analyses of medical records in the field of topical administration of FNS in the treatment of AGA. Overall, results from studies looking at the efficiency and effects of topical finasteride in male and female pattern alopecia show that finasteride is not inferior to systemic treatment.

The research shows that topical FNS may be safe for individuals who want to avoid systemic adverse effects, despite the low number of early data points on its usage. This may be particularly essential for the female androgenetic alopecia population, as systemic FNS is not permitted in this group because of hormonal inhibition, and it is a category X medication during pregnancy. Topical FNS may be used similarly to topical retinoid derivatives (category C) and its parent systemic medication, isotretinoin (category X). The topical version of finasteride is thought to be largely risk-free for use in pregnant women if the benefits outweigh the risks [40].

The vehicle, concentration, administration schedule, and frequency are only a few of the challenges that the relevant FNS research that is now accessible presents. The correct formulation of the vehicle impacts the pharmacokinetic and pharmacodynamic characteristics, dissolubility, strength, efficacy, pharmacodynamic interactions, and excretion. The administration of topical medications for any dermatological manifestation is a fiercely disputed issue. The composition of the vehicle likely affects the potency of topical finasteride preparations for treating AGA. The quantity of topical FNS formulations tested in gels and solutions varied, and all of them promoted hair growth [40].

The entire potential of topical FNS can only be understood with further study elucidating the best drug delivery mechanism, perfect concentration and frequency of medication administration, the adverse impact profile, and usage in other hair loss conditions.

## Conclusions

AGA is a chronic, crippling illness that adversely affects patients' quality of life and leads to significant psychological morbidity. The use of topical FNS for treating AGA has yielded encouraging preliminary results. Topical FNS may have therapeutic promise in managing AGA while limiting undesirable systemic side effects related to oral usage. Compared to systemic FNS, topical FNS does not seem inferior for hair regeneration. Topical finasteride alone may not be as successful as combination therapy using topical FNS in addition to MNX, dutasteride, or both. Despite its effectiveness and absence of adverse effects, topical finasteride is not frequently used. This is presumably a result of the dearth of investigations supported by evidence. The expense of compounding topical FNS may or may not deter patients from obtaining therapy. More research is needed to ascertain the efficacy of long-term hair regrowth, therapeutic safety, cost-effectiveness, patient tolerance, and satisfaction, given the advantages of topical finasteride in androgenetic alopecia patients.
